# Randomisierte, kontrollierte Studie zur palliativen Radiotherapie von Kopf-Hals-Tumoren – Herausforderungen bleiben bestehen

**DOI:** 10.1007/s00066-020-01672-2

**Published:** 2020-08-21

**Authors:** Alexander Fabian, David Krug, Jürgen Dunst

**Affiliations:** Klinik für Strahlentherapie, Universitätsklinikum Schleswig-Holstein (UKSH), Campus Kiel, Arnold-Heller-Str. 3, 24105 Kiel, Deutschland

## Hintergrund

In der palliativen Radiotherapie von Kopf-Hals-Tumoren findet eine Vielzahl unterschiedlicher Regime Verwendung. Das optimale Regime ist unklar. Aufgrund der schlechten Prognose müssen Therapiedauer, biologisch effektive Dosis und potenzielle Akut- und Spättoxizität gut gegeneinander abgewogen werden. Eine niederländische randomisierte Studie von Al-Mamgani und Kollegen verglich in diesem Kontext zwei Radiotherapieregime.

## Patienten und Methodik

Es handelt sich um eine zweiarmige Phase-III-Studie an 6 niederländischen Zentren. Einschlusskriterien waren: lokal fortgeschrittenes oder fernmetastasiertes Plattenepithelkarzinom des Oropharynx, Hypopharynx oder Larynx, ein guter Allgemeinzustand (ECOG 0–2) und keine Indikation für eine kurativ intendierte Lokaltherapie. Ein relevantes Ausschlusskriterium war eine antineoplastische Vorbehandlung des Kopf-Hals-Tumors. Die Patienten wurden 1:1 randomisiert. „Arm 1“ bestand aus einem Kurzzeitregime mit 6 Fraktionen von 6 Gy zweimal wöchentlich bis 36 Gy Gesamtdosis. „Arm 2“ war ein Langzeitregime mit 16 Fraktionen von 3,125 Gy viermal wöchentlich bis 50 Gy Gesamtdosis. In beiden Armen erfolgte eine intensitätsmodulierte oder volumetrisch modulierte (Rotations‑)Bestrahlung des makroskopischen Tumorvolumens, ggf. einschließlich von Lymphknotenmetastasen, mit einem CTV-Saum von 10 mm und einem PTV-Saum von 3 bis 5 mm ohne Behandlung elektiver Volumina. Primärer Endpunkt war die Zeit bis zur lokoregionären Progression. Die Autoren postulierten eine Verlängerung der Zeit bis zur lokoregionären Progression um mindestens 10 Wochen zugunsten von „Arm 2“. Sekundäre Endpunkte waren unter anderem das progressionsfreie Überleben, das Gesamtüberleben, die Toxizitätslast sowie die gesundheitsbezogene Lebensqualität. Die Fallzahlkalkulation mündete in 300 geplanten Patienten.

## Ergebnisse

In knapp 2 Jahren wurden 34 Patienten rekrutiert und randomisiert. Die Daten von 18 Patienten konnten in „Arm 1“ und von 14 Patienten in „Arm 2“ analysiert werden. Die Studie wurde aufgrund geringer Rekrutierung frühzeitig geschlossen. Alle Statistiken sind daher deskriptiv bzw. hypothesengenerierend. Die lokoregionäre Kontrolle betrug nach einem Jahr 57 % in „Arm 1“ und 69 % in „Arm 2“ (*p* = 0,45). Die mediane Zeit bis zur lokoregionären Progression war in beiden Armen noch nicht erreicht. Während das mediane progressionsfreie Überleben 5 Monate beziehungsweise 8 Monate betrug, lag das mediane Gesamtüberleben in „Arm 1“ bei knapp 9 Monaten und in „Arm 2“ bei knapp 15 Monaten (*p* = 0,2). Patienten in „Arm 1“ hatten mit 17 % gegenüber 57 % im „Arm 2“ signifikant weniger Grad-3-Toxizität, maßgeblich bedingt durch eine geringer ausgeprägte Mukositis. Die Mukositis führte bei 4 Patienten im „Arm 2“ zu einem frühzeitigen Therapieabbruch. In „Arm 1“ brach lediglich ein Patient die Therapie frühzeitig ab. Die gesundheitsbezogene Lebensqualität unterschied sich in beiden Armen nicht maßgeblich. Beide Arme zeigten einen Trend zu einer gebesserten allgemeinen gesundheitsbezogenen Lebensqualität (EORTC QLQ-C30) sowie zu gebesserten Schluckbeschwerden (EORTC QLQ-H&N35) und gebessertem bzw. stabilem Schmerzgeschehen (EQ-5D-5L VAS).

## Schlussfolgerungen der Autoren

Die vorzeitig abgebrochene Studie erlaubt keine zweifelsfreien Aussagen zur klinischen Überlegenheit eines der beiden Regime. Aufgrund der geringeren Toxizitätslast und statistisch nicht signifikant unterschiedlichen onkologischen Ergebnisse könnte das Kurzzeitregime (6 × 6 Gy) jedoch gegenüber dem Langzeitregime (16 × 3,125 Gy) favorisiert werden.

## Kommentar

Patienten mit einem Kopf-Hals-Tumor ohne kurative Option überleben im Median weniger als ein Jahr [1]. Selbst eine palliative Erstlinientherapie mit Chemo- und Immuntherapie konnte dies trotz der strengen Patientenselektion nur auf 13 bzw. 15 Monate verbessern [2]. Zusätzlich schränken Symptome wie Schmerzen, Blutungen und Schluckbeschwerden die Lebensqualität stark ein [3]. Häufig erfolgt eine palliative Radiotherapie in diesem Kontext [4]. Das optimale Radiotherapieregime ist jedoch unklar. Letzteres liegt zum Teil an den heterogenen Patientenkollektiven, die sich bezüglich Allgemeinzustand, Symptomatik, Tumorausdehnung und Indikation für die palliative Therapieintention deutlich unterscheiden (primär metastasierter vs. lokal weit fortgeschrittener Tumor vs. Komorbiditäten) [5]. Dies erfordert eine individualisierte Therapieplanung. Bislang fehlten daher auch randomisierte, kontrollierte Studien, um zu einer allgemein verbindlichen Vorgehensweise zu kommen. Al-Mamgani und Kollegen sind in ihren Bemühungen zu beglückwünschen, diese Lücke zu schließen. Ihre randomisierte Phase-III-Studie verglich ein Kurzzeitregime (6 × 6 Gy) mit einem Langzeitregime (16 × 3,125 Gy) zur palliativen Radiotherapie von Kopf-Hals-Tumoren. Mehrere Aspekte lassen es lohnenswert erscheinen, diese Studie näher zu reflektieren.

Erstens wurde die Studie frühzeitig abgebrochen. Anstatt der insgesamt 300 avisierten Patienten rekrutierten 6 niederländische Studienzentren in knapp 2 Jahren lediglich 34 Patienten. Dies veranschaulicht deutlich, wie schwierig es ist, in dieser Therapiesituation randomisierte strahlentherapeutische Studien durchzuführen, und könnte ein Spiegel der großen Heterogenität bei begrenzter Fallzahl sein.

Zweitens übertrifft das erreichte mediane Gesamtüberleben von 9 bzw. 15 Monaten teils deutlich das Gesamtüberleben der meisten bisher publizierten Kollektive mit nur ca. 6 Monaten [6]. Der gute Allgemeinzustand der eingeschlossenen Patienten (75 % ECOG 0–1; 25 % ECOG 2) könnte dies begründen; er deckt sich allerdings nur zum Teil mit unseren eigenen alltäglichen klinischen Erfahrungen. Ein weiterer Gesichtspunkt: Eine simultane Systemtherapie zur Radiotherapie war im Protokoll zwar nicht vorgesehen; inwieweit jedoch eine anschließende Chemo- und/oder Immuntherapie eingesetzt wurde und das Überleben beeinflusst haben könnte, wird in der Publikation nicht berichtet.

Auch wenn die statistische Auswertung bei 34 Patienten rein deskriptiv ist, zeigte sich drittens zwar ein numerischer Vorteil im Langzeitregime bezüglich lokaler Kontrolle und Gesamtüberleben, dieser war jedoch statistisch nicht signifikant. Demgegenüber bestand eine signifikant höhere Toxizitätslast im Langzeitregime, die auch zu mehr Therapieabbrüchen führte. Sowohl die onkologischen Endpunkte als auch die Toxizitätslast stehen im Einklang mit einarmigen Studien, die entweder ein Kurzzeit- oder ein Langzeitregime untersuchten. Fortin und Kollegen untersuchten beispielsweise 5 Fraktionen von 5 Gy an aufeinander folgenden Werktagen bei intensitätsmodulierter Radiotherapietechnik [7]. Das mediane Gesamtüberleben von 33 Patienten lag bei 6,5 Monaten, während lediglich 7 % eine Radiomukositis über Grad 2 aufwiesen. In einer einarmigen Vorläuferstudie untersuchten Al-Mamgani und Kollegen das Langzeitregime mit 16 Fraktionen von 3,215 Gy, jedoch ohne intensitätsmodulierte Radiotherapietechnik [8]. Das mediane Überleben von 158 Patienten lag hier bei 17 Monaten. Eine akute Radiomukositis über Grad 2 lag bei 65 % der Patienten vor. Der augenfällige Unterschied im medianen Überleben könnte unter anderem durch einen deutlich höheren Anteil an Patienten mit fernmetastasierten Erkrankungen in der Studie von Fortin und Kollegen bedingt sein (knapp 25 % vs. 9 %).

Viertens waren „patient-reported outcomes“ zur Erhebung der gesundheitsbezogenen Lebensqualität lediglich ein sekundärer Endpunkt. In der palliativen Situation mit begrenzter Lebenserwartung sollte aber die gesundheitsbezogene Lebensqualität im Vordergrund stehen [9]. Bislang gibt es jedoch kaum Studien in diesem Kontext, die die gesundheitsbezogene Lebensqualität durch eine validierte Erhebung als primären Endpunkt einsetzen. Letzteres wäre wichtig, um den Wert der palliativen Radiotherapie von Kopf-Hals-Tumoren zu sichern, der zum Teil bereits infrage gestellt worden ist [10]. Aus diesem Grund haben wir eine prospektive multizentrische Beobachtungsstudie mit dem primären Endpunkt der gesundheitsbezogenen Lebensqualität initiiert (DRKS00021197).

## Fazit

Zusammenfassend liegen die Herausforderungen in der palliativen Radiotherapie von Kopf-Hals-Tumoren in dem heterogenen Patientenkollektiv und der individuell abzuwägenden Balance zwischen Toxizität und antineoplastischer Effektivität. Während Patienten in reduziertem Allgemeinzustand von einem Kurzzeitregime mit geringerer Toxizitätslast profitieren könnten, könnte fitteren Patienten eine langfristigere Tumorkontrolle durch ein Langzeitregime ermöglicht werden. Der Fokus sollte dabei insbesondere auf einer Stabilisierung oder Verbesserung der gesundheitsbezogenen Lebensqualität liegen, was durch weitere Studien belegt werden muss.

*Alexander Fabian, David Krug und Jürgen Dunst, Kiel*
